# Syntheses and crystal structures of 2-methyl-1,1,2,3,3-penta­phenyl-2-sila­propane and 2-methyl-1,1,3,3-tetra­phenyl-2-silapropan-2-ol

**DOI:** 10.1107/S2056989019011265

**Published:** 2019-08-23

**Authors:** Alexandra Williams, Michelle Brown, Richard J. Staples, Shannon M. Biros, William R. Winchester

**Affiliations:** a Department of Chemistry, Grand Valley State University, 1 Campus Dr., Allendale, MI 49401, USA; b Center for Crystallographic Research, Michigan State University, Department of Chemistry and Chemical Biology, East Lansing, MI 48824, USA

**Keywords:** crystal structure, silanol, steric hindrance, di­phenyl­meth­yl, benzhydr­yl, O—H⋯π inter­action, C—H⋯π inter­action

## Abstract

The sterically hindered silicon compound 2-methyl-1,1,2,3,3-penta­phenyl-2-sila­propane, C_33_H_30_Si (**I**), was prepared *via* the reaction of two equivalents of di­phenyl­methyl­lithium (benzhydryllithium) and di­chloro­methyl­phenyl­silane. This bis­benzhydryl-substituted silicon compound was then reacted with tri­fluoro­methane­sulfonic acid, followed by hydrolysis with water to give the silanol 2-methyl-1,1,3,3-tetra­phenyl-2-silapropan-2-ol, C_27_H_26_OSi (**II**). Key geometric features for **I** are the Si—C bond lengths that range from 1.867 (2) to 1.914 (2) Å and a τ_4_ descriptor for fourfold coordination around the Si atom of 0.97 (indicating a nearly perfect tetra­hedron). Key geometric features for compound **II** include Si—C bond lengths that range from 1.835 (4) to 1.905 (3) Å, a Si—O bond length of 1.665 (3) Å, and a τ_4_ descriptor for fourfold coordination around the Si atom of 0.96. In compound **II**, there is an intra­molecular C—H⋯O hydrogen bond present. In the crystal of **I**, mol­ecules are linked by two pairs of C—H⋯π inter­actions, forming dimers that are linked into ribbons propagating along the *b*-axis direction. In the crystal of **II**, mol­ecules are linked by C—H⋯π and O—H⋯π inter­actions that result in the formation of ribbons that run along the *a*-axis direction.

## Abstract 2

The structures of two sterically hindered silicon compounds feature inter­molecular C—H⋯π inter­actions in the solid state. The silapropan-2-ol compound also features an inter­molecular O—H⋯π inter­action.

## Chemical context   

**Figure 1:**
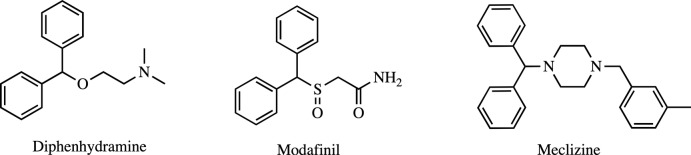
The benzhydryl group in some representative medicinal compounds.

The benzhydryl substituent and its derivatives occur in many medicinal compounds, for example: diphenhydramine, modafinil and meclizine (Fig. 1 ▸). The addition of the benzhydryl group to a drug significantly increases its lipophilicity and the two aromatic rings add electron density and bulk. There is an active field looking at the switching of silicon for carbon to discover new medicinal compounds and there have been several recent publications and reviews in the area (Franz & Wilson, 2013 ▸; Geyer *et al.*, 2015 ▸; Ramesh & Reddy, 2018 ▸; Tacke & Doerrich, 2016 ▸). It seemed to us that another option is to replace the sulfoxide group with a silanol, in which the silicon has a size that is similar to sulfur and the alcohol will occupy the space of the sulfoxide oxygen. The conversion of a phenyl­silane to a silanol by the reaction with tri­fluoro­methane­sulfonic acid followed by hydrolysis has been used previously (Kira *et al.*, 2007 ▸; Shainyan *et al.*, 2017 ▸), and worked well for the introduction of the silanol in compound **II**, silanol 2-methyl-1,1,3,3-tetra­phenyl-2-silapropan-2-ol.
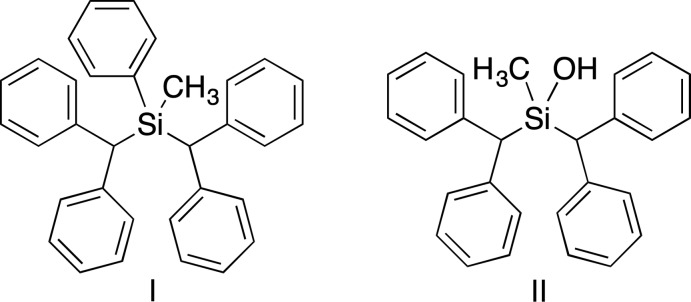


The steric bulk of the benzhydryl group has been used to advantage in several silyl reagents. It has been reported that the benzhydryldi­methyl­silylgroup is readily synthesized and undergoes facile oxidation with hydrogen peroxide to form alcohols (Peng & Woerpel, 2001 ▸). Yoshida and coworkers have started with a tris­(di­phenyl­meth­yl)silane and further substituted aromatic rings using electrophilic aromatic substitution to produce the sterically demanding TEDAMS group (Terao *et al.*, 2010 ▸). Unno *et al.* (2006 ▸) have addressed the influence of bulky silyl groups on the ability of silanols to hydrogen bond, and found that (*i*-Pr_3_Si)_3_SiOH exists as a monomer while (*t*-BuMe_2_Si)_3_SiOH is a hydrogen-bonded dimer. Our observation that compound **II** is monomeric indicates that the silicon atom is very hindered by the presence of the two benzhydryl groups.

## Structural commentary   

**Figure 2:**
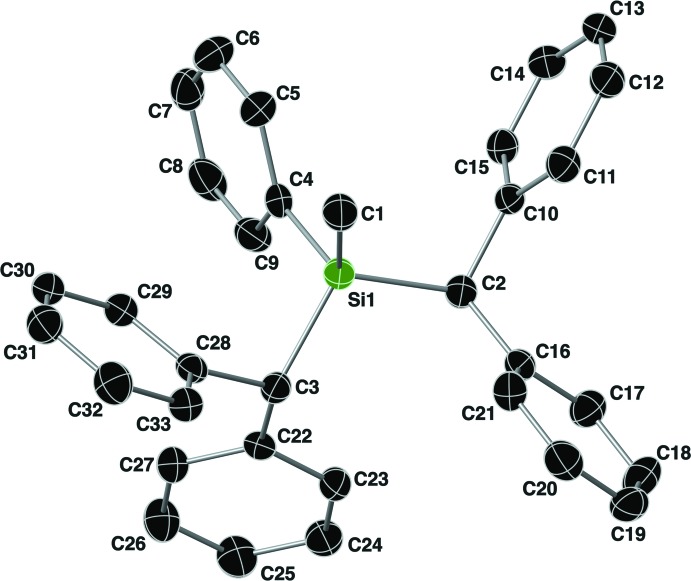
The mol­ecular structure of compound **I**, with the atom-labeling scheme. Displacement ellipsoids are drawn at the 40% probability level. For clarity, the hydrogen atoms have been omitted.

The mol­ecular structure of compound **I** is shown in Fig. 2 ▸. The Si—C bond lengths range from 1.867 (2) to 1.914 (2) Å, with the Si—C1 bond to the methyl group being the shortest. The τ_4_ descriptor for fourfold coordination around Si1 is 0.97, indicating a nearly perfect tetra­hedral geometry around this silicon atom (where 0 = square planar, 0.85 = trigonal pyramidal, and 1 = tetra­hedral; Yang *et al.*, 2007 ▸). The Si1—C1 bond and aromatic ring (C4–C9) are nearly co-planar with a C1—Si1—C4—C5 torsion angle of 12.2 (2)°. The orientation of the benzhydryl group bonded to C2 is such that when the mol­ecule is viewed down the C2—Si1 bond the methyl group (C1) is *anti* to H2 (torsion angle C1—Si1—C2—H2 is 169°), with the aromatic rings *gauche*. For the benzhydryl group containing C3, the hydrogen atom H3 is *gauche* to the methyl group (C1) with a C1—Si1—C3—H3 torsion angle of 69°, with the aromatic ring (C22–C27) occupying the *anti* position.

**Figure 3:**
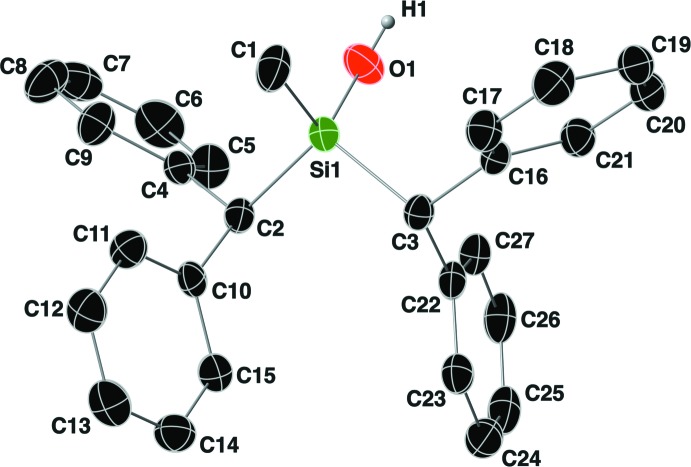
The mol­ecular structure of compound **II**, with the atom-labeling scheme. Displacement ellipsoids are drawn at the 40% probability level. For clarity, the C-bound hydrogen atoms have been omitted.

The mol­ecular structure of compound **II** is shown in Fig. 3 ▸. The Si—C bond lengths range from 1.835 (4) to 1.905 (3) Å, with an Si—O bond length of 1.665 (3) Å. The τ_4_ descriptor for fourfold coordination around Si1 is 0.96, again indicating an almost perfect tetra­hedral geometry around this silicon atom. The orientation of the C2 benzhydryl group is such that the hydrogen atom H2 is *anti* to the methyl group (C1) with a C1—Si1—C2—H2 torsion angle of −165°. For the benzhydryl group containing C3, the hydrogen atom H3 is again *gauche* to the methyl group (C1) with a C1—Si1—C3—H3 torsion angle of 55°, and the aromatic ring C22–C27 occupies the *anti* position. An intra­molecular C—H⋯O hydrogen bond is present between H27 and O1 with an H⋯*A* distance of 2.55 Å (Table 2 ▸).

## Supra­molecular features   

**Figure 4:**
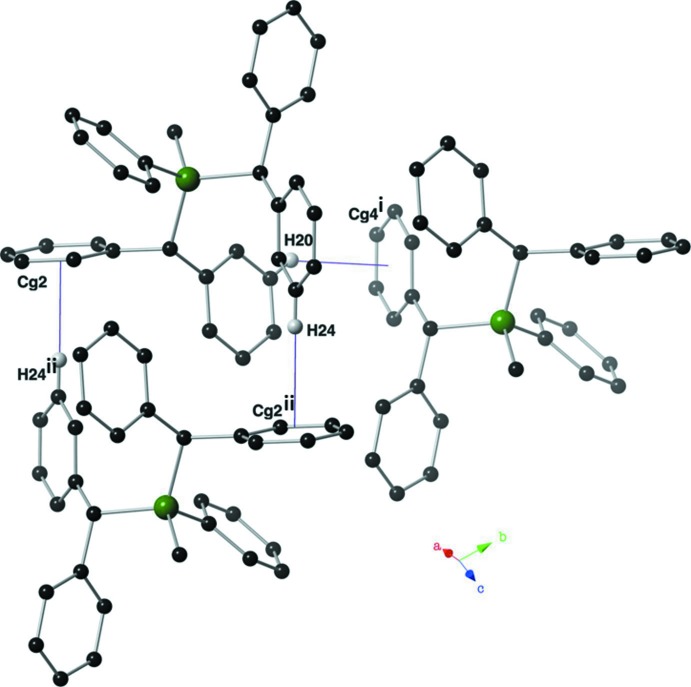
Inter­molecular C—H⋯π inter­actions present in the crystal of compound **I**; see Table 1 ▸ for details. Only hydrogen atoms H24 and H20 are shown for clarity, and the C—H⋯π inter­actions are depicted as purple lines. Symmetry codes: (i) −*x* + 2, −*y*, −*z*; (ii) −*x* + 2, −*y* + 1, −*z*.

**Figure 5:**
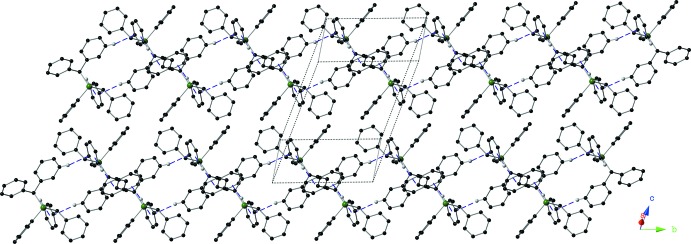
The crystal packing of compound **I**, viewed along the *a*-axis, showing the supra­molecular ribbons formed by inter­molecular C—H⋯π inter­actions (Table 1 ▸; shown as dashed purple lines). Only hydrogen atoms H20 and H24 are shown for clarity.

In the crystal of **I**, mol­ecules are linked by two pairs of inter­molecular C—H⋯π inter­actions involving inversion-related compounds (Fig. 4 ▸ and Table 1 ▸). The result of these inter­actions is the formation of dimers that are linked to form ribbons along the *b*-axis direction (Fig. 5 ▸).

**Figure 6:**
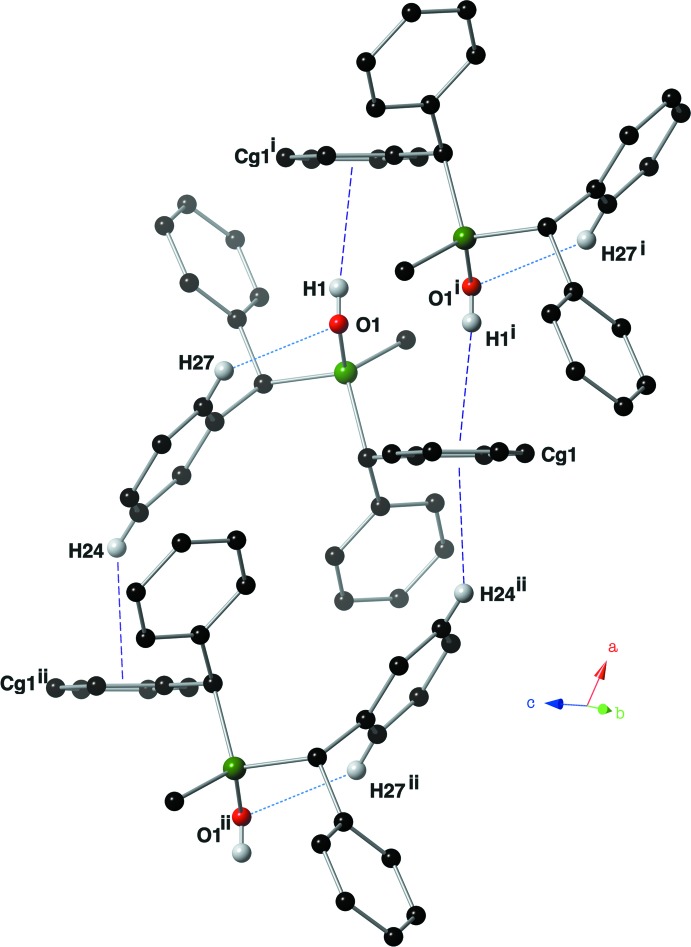
Intra­molecular hydrogen bond (blue dotted lines) and inter­molecular C—H⋯π and O—H⋯π inter­actions (Table 2 ▸; purple dashed lines) present in the crystal of compound **II**. For clarity, only hydrogen atoms H1, H24 and H27 have been included. Symmetry codes: (i) −*x* + 1, −*y* + 1, −*z*; (ii) −*x*, −*y* + 1, −*z*.

**Figure 7:**
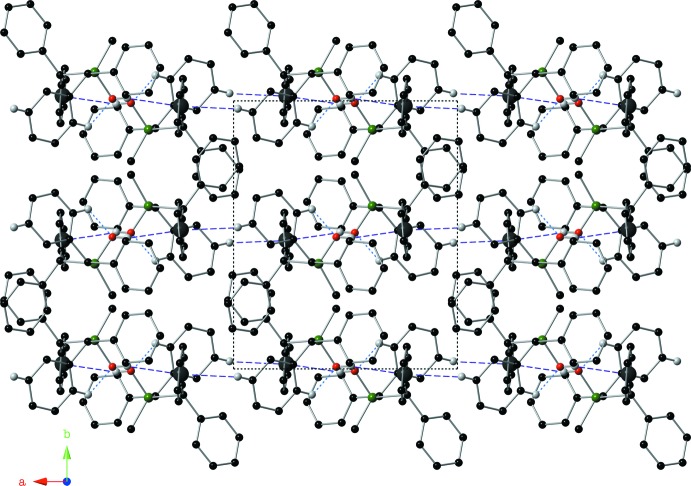
The crystal packing of compound **II**, viewed along the *c*-axis, showing the supra­molecular ribbons formed by O—H⋯π and C—H⋯π inter­actions (Table 2 ▸). For clarity, only hydrogen atoms H1, H24 and H27 have been included.

In the crystal of **II**, inversion-related mol­ecules are linked by a pair of O—H⋯π inter­actions, forming dimers (Table 2 ▸, Fig. 6 ▸). Similar inter­actions between aryl groups and OH groups in silanols have been reported previously (Al-Juaid *et al.*, 1992 ▸). In the crystal of **II**, the dimers are linked by a pair of C—H⋯π inter­actions (Table 2 ▸), so forming ribbons that propagate along the *a*-axis direction (Fig. 7 ▸).

## Database survey   

A search of the Cambridge Structural Database (CSD, Version 5.40, May 2019; Groom *et al.*, 2016 ▸) gave only one hit for a structure in which a silicon atom is bonded to two benz­hydryl groups, *viz*. bis­(di­ethyl­amino)bis­(di­phenyl­meth­yl)silane (CSD refcode YEPTUI; Huppmann, *et al.*, 1994 ▸). In this compound, the silicon atom is also bonded to two di­ethyl­amino groups. There are four other structures in the CSD with a silicon atom bonded to one benz­hydryl group and a different alkyl group (this count excludes organometallic compounds). These compounds include, *tert*-butyl 1′-acetyl-4-[(di­phenyl­meth­yl)(dimeth­yl)sil­yl]-5′-fluoro-2′-oxo-1′,2′-di­hydro­spiro­[cyclo­pentane-1,3′-indole]-2-carboxyl­ate (SOSZIL; Ball-Jones *et al.*, 2014 ▸), (3*S*,4*R*,5*S*)-4-[dimeth­yl(di­phenyl­meth­yl)sil­yl]-5-{[dimeth­yl(phen­yl)sil­yl]meth­yl}-3-meth­yl-tetra­hydro­furan-2-one (XICWUB; Peng & Woerpel, 2001 ▸), diphen­yl(tri­methyl­sil­yl)methane (MOQWIY; Hill & Hitchcock, 2002 ▸) and diphen­yl[*t*-but­yl(dimeth­yl)sil­yl]methane (MOQWEU; Hill & Hitchcock, 2002 ▸). This search revealed zero structures in the CSD that contained a silanol group where the silicon atom is bonded to a benz­hydryl group. However, the related structures (tri­phenyl­meth­yl)silanetriol acetone solvate (GAWVUW; Kim, *et al.*, 2005 ▸) and (tri­phen­yl­meth­yl)silanetriol tetra­hydro­furan solvate (BAVQOF; Yoo, *et al.*, 2001 ▸) are both silanetriols that bear a trityl group (–CPh_3_) coordinated to the central silicon atom.

## Synthesis and crystallization   

**Synthesis of 2-methyl-1,1,2,3,3-penta­phenyl-2-sila­propane (I)**: Di­phenyl­methane (1.68 g, 10 mmol) was added to an oven-dried, argon-flushed 100 ml Schlenk flask along with a magnetic stirbar. Anhydrous tetra­hydro­furan (10 ml) was then added to the flask to dissolve the solid and the solution was cooled to 273 K. After the solution had cooled for 10 min, *n*-butyl­lithium (6.25 ml, 1.6 *M* in hexa­nes, 10 mmol) was added and the solution was stirred for 1 h. The reaction mixture was then cooled further to 195 K and di­chloro­methyl­phenyl­silane was added (0.955g, 5 mmol). After warming to room temperature and stirring for 12 h, the solution was poured into hexa­nes (20 ml) and the organic layer was washed with water (20 ml), dilute hydro­chloric acid (3 *N*, 10 ml), water (10 ml) and finally brine (10 ml). The hexa­nes solution was dried over sodium sulfate, filtered and concentrated *in vacuo*. The product was purified by dissolving it in 20 ml hexane, cooling to 195 K and isolating the white crystals by filtration. The crystals were then washed with pentane and dried *in vacuo* (2.1 g, 93% yield). Colorless block-like crystals suitable for analysis by X-ray diffraction were grown by recrystallization of compound **I** (0.1 g) from hexa­nes (2 ml) with heating (0.08 g isolated yield). FT–IR (*ν*, cm^−1^): 3057, 3019, 2869, 1597, 1493, 696; ^1^H NMR (400 MHz, chloro­form-*d*) δ 0.39 (*s*, 3H), 3.87 (*s*, 2H), 6.8–7.4 (*m*, 25H); ^13^C NMR (101 MHz, chloro­form-*d*) δ −4.77, 42.70, 125.28, 125.67, 127.37, 128.14, 128.49, 129.21, 129.46, 129.81, 134.62, 135.99, 142.02, 142.14; ^29^Si NMR (79 MHz, chloro­form-*d*) δ −3.12.

**Synthesis of 2-methyl-1,1,3,3-tetra­phenyl-2-silapropan-2-ol (II)**: Bis(di­phenyl­meth­yl)methyl­phenyl­silane (0.455 g, 1.0 mmol) was added to an oven-dried, argon-flushed 50 ml Schlenk flask along with a stirbar. Anhydrous toluene (5 ml) was added to dissolve the solid and the solution was cooled to 273 K. Tri­fluoro­methane­sulfonic acid was weighed in a vial (150 mg, 1 mmol) and then added to the Schlenk flask using a Pasteur pipette, at which point the solution went from colorless to a bright yellow. The solution was stirred for 2 h at room temperature after which time the solution went from cloudy to clear. At this point a mixture of water (40 mg, 2.2 mmol) and tri­ethyl­amine (200 mg, 2.0 mmol) in ether (2 ml) was prepared and added to the rapidly stirring solution of the triflate in toluene, which caused the yellow solution to immediately turn colorless. After stirring for 1 h, the mixture was poured into hexa­nes (20 ml) and the organic layer was washed with water (20 ml), dilute hydro­chloric acid (3 *N*, 10 ml), water (10 ml) and finally brine (10 ml). The hexa­nes solution was dried over sodium sulfate, filtered and the solvent was removed *in vacuo*. The crude product was then dissolved in 5 ml hexane and cooled to 195 K. The white crystals were isolated by vacuum filtration, washed with pentane and dried *in vacuo* (319 mg, 81% yield). Colourless block-like crystals suitable for analysis by X-ray diffraction were grown by recrystallization of compound **II** (0.4 g) from hexa­nes (5 ml) with heating (0.3 g isolated yield, m.p. (uncorrected) 375.8–376.2 K). FT–IR (*ν*, cm^−1^): 3591, 3057, 3024, 1597, 1491, 696; ^1^H NMR (400 MHz, chloro­form-*d*) δ 0.15 (*s*, 3H), 3.49 (*s*, 2H), 7.22 (*m*, 20H). ^13^C NMR (101 MHz, chloro­form-*d*) δ −2.34, 44.44, 125.64, 125.68, 128.62, 129.06, 129.28, 141.20, 141.43. ^29^Si NMR (79 MHz, chloro­form-*d*) δ 7.98.

## Refinement   

Crystal data, data collection and structure refinement details are summarized in Table 3 ▸. For both compounds, the hydrogen atoms bonded to carbon atoms were placed in calculated positions and refined as riding: C—H = 0.95–1.00 Å with *U*_iso_(H) = 1.5*U*_eq_(C-meth­yl) and 1.2*U*_eq_(C) for other H atoms in compound **I**, and C—H = 0.93–0.98 Å with 1.2*U*_eq_(C) in compound **II**. The hydrogen atom bonded to O1 (H1) in compound **II** was located in an electron-density difference map and freely refined.

## Supplementary Material

Crystal structure: contains datablock(s) Global, II, I. DOI: 10.1107/S2056989019011265/su5509sup1.cif

Structure factors: contains datablock(s) I. DOI: 10.1107/S2056989019011265/su5509Isup2.hkl

Structure factors: contains datablock(s) II. DOI: 10.1107/S2056989019011265/su5509IIsup3.hkl

Supporting information file. DOI: 10.1107/S2056989019011265/su5509Isup4.cml

Supporting information file. DOI: 10.1107/S2056989019011265/su5509IIsup5.cml

CCDC references: 1946744, 1946743

Additional supporting information:  crystallographic information; 3D view; checkCIF report

## Figures and Tables

**Table 1:** Hydrogen-bond geometry (Å, °) for **I** [Chem scheme1] *Cg*2 and *Cg*4 are the centroids of the C10–C15 and C22–C27 rings, respectively.

*D*—H⋯*A*	*D*—H	H⋯*A*	*D*⋯*A*	*D*—H⋯*A*
C20—H20⋯*Cg*4^i^	0.95	2.94	3.716 (2)	140
C24—H24⋯*Cg*2^ii^	0.95	2.75	3.696 (2)	175

Symmetry codes: (i) 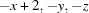; (ii) 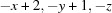.

**Table 2:** Hydrogen-bond geometry (Å, °) for **II** [Chem scheme1] *Cg*1 is the centroid of the C4–C9 ring.

*D*—H⋯*A*	*D*—H	H⋯*A*	*D*⋯*A*	*D*—H⋯*A*
C27—H27⋯O1	0.93	2.55	3.237 (4)	131
O1—H1⋯*Cg*1^i^	0.75 (8)	2.70 (7)	3.416 (3)	162 (7)
C24—H24⋯*Cg*1^ii^	0.93	2.86	3.582 (4)	135

Symmetry codes: (i) 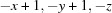; (ii) 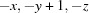.

**Table 3:** Experimental details

	**I**	**II**
Crystal data		
Chemical formula	C_33_H_30_Si	C_27_H_26_OSi
*M*_r_	454.66	394.57
Crystal system, space group	Triclinic, *P* 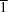	Monoclinic, *P*2_1_/*c*
Temperature (K)	173	173
*a*, *b*, *c* (Å)	10.3879 (7), 10.5037 (7), 13.6350 (9)	11.8576 (5), 13.2995 (6), 14.3948 (6)
α, β, γ (°)	68.8212 (7), 70.6364 (7), 84.7947 (8)	90, 110.363 (3), 90
*V* (Å^3^)	1308.06 (15)	2128.20 (16)
*Z*	2	4
Radiation type	Mo *K*α	Cu *K*α
μ (mm^−1^)	0.11	1.08
Crystal size (mm)	0.21 × 0.21 × 0.17	0.10 × 0.09 × 0.08

Data collection		
Diffractometer	Bruker APEXII CCD	Bruker APEXII CCD
Absorption correction	Multi-scan (*SADABS*; Bruker, 2013 ▸)	Multi-scan (*SADABS*; Bruker, 2013 ▸
*T*_min_, *T*_max_	0.695, 0.745	0.624, 0.754
No. of measured, independent and observed [*I* > 2σ(*I*)] reflections	16773, 4803, 3734	11901, 4113, 2414
*R*_int_	0.036	0.096
(sin θ/λ)_max_ (Å^−1^)	0.603	0.617

Refinement		
*R*[*F* ^2^ > 2σ(*F* ^2^)], *wR*(*F* ^2^), *S*	0.045, 0.120, 1.08	0.062, 0.171, 0.97
No. of reflections	4803	4113
No. of parameters	308	267
H-atom treatment	H-atom parameters constrained	H atoms treated by a mixture of independent and constrained refinement
Δρ_max_, Δρ_min_ (e Å^−3^)	0.34, −0.22	0.29, −0.36

Computer programs: *APEX2* and *SAINT* (Bruker, 2013 ▸), *SHELXS* (Sheldrick, 2008 ▸), *SHELXL* (Sheldrick, 2015 ▸), *OLEX2* (Dolomanov *et al.*, 2009 ▸; Bourhis *et al.*, 2015 ▸) and *CrystalMaker* (Palmer, 2007 ▸).

## supplementary crystallographic information

### 2-Methyl-1,1,2,3,3-pentaphenyl-2-silapropane (I) Crystal data

**Table:**

C_33_H_30_Si	*Z* = 2
*M**_r_* = 454.66	*F*(000) = 484
Triclinic, *P*1	*D*_x_ = 1.154 Mg m^−^^3^
*a* = 10.3879 (7) Å	Mo *K*α radiation, λ = 0.71073 Å
*b* = 10.5037 (7) Å	Cell parameters from 6620 reflections
*c* = 13.6350 (9) Å	θ = 2.2–25.4°
α = 68.8212 (7)°	µ = 0.11 mm^−^^1^
β = 70.6364 (7)°	*T* = 173 K
γ = 84.7947 (8)°	Block, colourless
*V* = 1308.06 (15) Å^3^	0.21 × 0.21 × 0.17 mm

### 2-Methyl-1,1,2,3,3-pentaphenyl-2-silapropane (I) Data collection

**Table:**

Bruker APEXII CCD diffractometer	3734 reflections with *I* > 2σ(*I*)
φ and ω scans	*R*_int_ = 0.036
Absorption correction: multi-scan (SADABS; Bruker, 2013)	θ_max_ = 25.4°, θ_min_ = 1.7°
*T*_min_ = 0.695, *T*_max_ = 0.745	*h* = −12→12
16773 measured reflections	*k* = −12→12
4803 independent reflections	*l* = −16→16

### 2-Methyl-1,1,2,3,3-pentaphenyl-2-silapropane (I) Refinement

**Table:**

Refinement on *F*^2^	Primary atom site location: structure-invariant direct methods
Least-squares matrix: full	Secondary atom site location: difference Fourier map
*R*[*F*^2^ > 2σ(*F*^2^)] = 0.045	Hydrogen site location: inferred from neighbouring sites
*wR*(*F*^2^) = 0.120	H-atom parameters constrained
*S* = 1.08	*w* = 1/[σ^2^(*F*_o_^2^) + (0.0521*P*)^2^ + 0.3536*P*] where *P* = (*F*_o_^2^ + 2*F*_c_^2^)/3
4803 reflections	(Δ/σ)_max_ < 0.001
308 parameters	Δρ_max_ = 0.34 e Å^−^^3^
0 restraints	Δρ_min_ = −0.22 e Å^−^^3^

### 2-Methyl-1,1,2,3,3-pentaphenyl-2-silapropane (I) Special details

**Table:**

Geometry. All esds (except the esd in the dihedral angle between two l.s. planes) are estimated using the full covariance matrix. The cell esds are taken into account individually in the estimation of esds in distances, angles and torsion angles; correlations between esds in cell parameters are only used when they are defined by crystal symmetry. An approximate (isotropic) treatment of cell esds is used for estimating esds involving l.s. planes.

### 2-Methyl-1,1,2,3,3-pentaphenyl-2-silapropane (I) Fractional atomic coordinates and isotropic or equivalent isotropic displacement parameters (Å^2^)

**Table:**

	*x*	*y*	*z*	*U*_iso_*/*U*_eq_	
Si1	0.75666 (5)	0.17147 (5)	0.27134 (4)	0.02998 (15)	
C1	0.58208 (18)	0.0942 (2)	0.31735 (16)	0.0399 (5)	
H1A	0.580675	0.040999	0.271655	0.060*	
H1B	0.516044	0.166936	0.309113	0.060*	
H1C	0.557892	0.034234	0.395435	0.060*	
C2	0.78889 (17)	0.32685 (18)	0.13751 (14)	0.0293 (4)	
H2	0.875549	0.371155	0.128137	0.035*	
C3	0.88432 (17)	0.03163 (18)	0.25388 (14)	0.0290 (4)	
H3	0.879419	0.013883	0.187902	0.035*	
C4	0.77121 (19)	0.23587 (18)	0.37890 (15)	0.0337 (4)	
C5	0.6560 (2)	0.2462 (2)	0.46416 (16)	0.0436 (5)	
H5	0.569129	0.220352	0.467114	0.052*	
C6	0.6643 (3)	0.2927 (2)	0.54426 (18)	0.0549 (6)	
H6	0.583678	0.298764	0.601188	0.066*	
C7	0.7882 (3)	0.3304 (2)	0.54221 (19)	0.0539 (6)	
H7	0.793896	0.361141	0.598221	0.065*	
C8	0.9045 (2)	0.3235 (2)	0.45856 (19)	0.0492 (6)	
H8	0.990631	0.350498	0.456297	0.059*	
C9	0.8961 (2)	0.2771 (2)	0.37750 (17)	0.0401 (5)	
H9	0.976882	0.273302	0.319904	0.048*	
C10	0.67921 (17)	0.43090 (18)	0.15447 (14)	0.0294 (4)	
C11	0.55994 (19)	0.4316 (2)	0.12873 (16)	0.0361 (4)	
H11	0.546285	0.365865	0.099890	0.043*	
C12	0.46085 (19)	0.5266 (2)	0.14451 (16)	0.0415 (5)	
H12	0.380019	0.525025	0.126874	0.050*	
C13	0.4791 (2)	0.6233 (2)	0.18562 (16)	0.0416 (5)	
H13	0.411325	0.688460	0.196376	0.050*	
C14	0.5969 (2)	0.6242 (2)	0.21100 (16)	0.0403 (5)	
H14	0.610168	0.690778	0.239220	0.048*	
C15	0.69603 (19)	0.52935 (19)	0.19580 (15)	0.0348 (4)	
H15	0.776475	0.531510	0.213801	0.042*	
C16	0.81390 (17)	0.30119 (18)	0.02966 (15)	0.0302 (4)	
C17	0.89389 (19)	0.3951 (2)	−0.06919 (16)	0.0395 (5)	
H17	0.929517	0.474662	−0.068264	0.047*	
C18	0.9227 (2)	0.3751 (2)	−0.16910 (17)	0.0481 (5)	
H18	0.977671	0.440845	−0.235724	0.058*	
C19	0.8724 (2)	0.2603 (2)	−0.17273 (17)	0.0458 (5)	
H19	0.894110	0.245561	−0.241102	0.055*	
C20	0.7903 (2)	0.1675 (2)	−0.07596 (18)	0.0454 (5)	
H20	0.753497	0.089032	−0.077590	0.054*	
C21	0.7611 (2)	0.1883 (2)	0.02404 (16)	0.0387 (5)	
H21	0.703758	0.123685	0.090102	0.046*	
C22	1.03391 (17)	0.06948 (17)	0.22632 (14)	0.0288 (4)	
C23	1.10054 (19)	0.16811 (19)	0.12395 (15)	0.0365 (5)	
H23	1.051484	0.211263	0.073556	0.044*	
C24	1.2365 (2)	0.2044 (2)	0.09431 (17)	0.0438 (5)	
H24	1.279369	0.272864	0.024624	0.053*	
C25	1.3101 (2)	0.1413 (2)	0.16568 (18)	0.0446 (5)	
H25	1.403699	0.165709	0.145408	0.054*	
C26	1.2463 (2)	0.0427 (2)	0.26653 (17)	0.0421 (5)	
H26	1.296597	−0.001657	0.315750	0.050*	
C27	1.10940 (19)	0.00747 (19)	0.29711 (15)	0.0341 (4)	
H27	1.066750	−0.060027	0.367444	0.041*	
C28	0.82851 (17)	−0.09864 (18)	0.35234 (15)	0.0300 (4)	
C29	0.80721 (19)	−0.10980 (19)	0.46160 (15)	0.0347 (4)	
H29	0.833986	−0.035807	0.475796	0.042*	
C30	0.74772 (19)	−0.2268 (2)	0.54981 (16)	0.0388 (5)	
H30	0.734851	−0.232753	0.623661	0.047*	
C31	0.7071 (2)	−0.3347 (2)	0.53092 (17)	0.0432 (5)	
H31	0.664258	−0.414051	0.591663	0.052*	
C32	0.7288 (2)	−0.3271 (2)	0.42349 (18)	0.0452 (5)	
H32	0.702693	−0.401861	0.409890	0.054*	
C33	0.78895 (19)	−0.20994 (19)	0.33547 (16)	0.0365 (4)	
H33	0.803491	−0.205565	0.261749	0.044*	

### 2-Methyl-1,1,2,3,3-pentaphenyl-2-silapropane (I) Atomic displacement parameters (Å^2^)

**Table:**

	*U*^11^	*U*^22^	*U*^33^	*U*^12^	*U*^13^	*U*^23^
Si1	0.0258 (3)	0.0328 (3)	0.0277 (3)	0.0011 (2)	−0.0077 (2)	−0.0073 (2)
C1	0.0296 (10)	0.0442 (11)	0.0373 (11)	−0.0004 (9)	−0.0088 (9)	−0.0059 (9)
C2	0.0258 (9)	0.0315 (10)	0.0295 (10)	−0.0010 (7)	−0.0094 (8)	−0.0086 (8)
C3	0.0280 (9)	0.0321 (10)	0.0258 (9)	0.0014 (7)	−0.0088 (7)	−0.0092 (8)
C4	0.0377 (10)	0.0305 (10)	0.0282 (10)	0.0086 (8)	−0.0126 (8)	−0.0050 (8)
C5	0.0459 (12)	0.0458 (12)	0.0319 (11)	0.0053 (10)	−0.0075 (9)	−0.0111 (9)
C6	0.0677 (16)	0.0552 (14)	0.0333 (12)	0.0104 (12)	−0.0060 (11)	−0.0173 (11)
C7	0.0861 (19)	0.0466 (13)	0.0412 (13)	0.0236 (12)	−0.0339 (13)	−0.0222 (11)
C8	0.0592 (14)	0.0445 (12)	0.0609 (15)	0.0170 (11)	−0.0375 (12)	−0.0249 (11)
C9	0.0383 (11)	0.0410 (11)	0.0459 (12)	0.0115 (9)	−0.0176 (9)	−0.0196 (10)
C10	0.0282 (9)	0.0312 (9)	0.0241 (9)	−0.0005 (7)	−0.0076 (7)	−0.0048 (8)
C11	0.0328 (10)	0.0414 (11)	0.0353 (11)	0.0007 (8)	−0.0139 (8)	−0.0122 (9)
C12	0.0290 (10)	0.0557 (13)	0.0368 (11)	0.0079 (9)	−0.0134 (9)	−0.0120 (10)
C13	0.0374 (11)	0.0489 (12)	0.0340 (11)	0.0151 (9)	−0.0098 (9)	−0.0141 (10)
C14	0.0447 (12)	0.0412 (11)	0.0372 (11)	0.0068 (9)	−0.0121 (9)	−0.0184 (9)
C15	0.0340 (10)	0.0409 (11)	0.0305 (10)	0.0027 (8)	−0.0136 (8)	−0.0113 (9)
C16	0.0256 (9)	0.0351 (10)	0.0304 (10)	0.0053 (8)	−0.0119 (8)	−0.0106 (8)
C17	0.0360 (11)	0.0473 (12)	0.0336 (11)	−0.0033 (9)	−0.0106 (9)	−0.0121 (9)
C18	0.0414 (12)	0.0656 (15)	0.0312 (11)	−0.0012 (11)	−0.0091 (9)	−0.0118 (10)
C19	0.0427 (12)	0.0668 (15)	0.0368 (12)	0.0137 (11)	−0.0168 (10)	−0.0274 (11)
C20	0.0543 (13)	0.0442 (12)	0.0526 (14)	0.0126 (10)	−0.0287 (11)	−0.0263 (11)
C21	0.0429 (11)	0.0366 (11)	0.0365 (11)	0.0002 (9)	−0.0163 (9)	−0.0094 (9)
C22	0.0272 (9)	0.0292 (9)	0.0303 (10)	0.0055 (7)	−0.0085 (8)	−0.0126 (8)
C23	0.0324 (10)	0.0384 (11)	0.0302 (10)	0.0056 (8)	−0.0082 (8)	−0.0052 (9)
C24	0.0336 (11)	0.0433 (12)	0.0379 (12)	−0.0025 (9)	−0.0027 (9)	−0.0028 (9)
C25	0.0310 (11)	0.0460 (12)	0.0513 (13)	−0.0058 (9)	−0.0122 (10)	−0.0101 (10)
C26	0.0371 (11)	0.0444 (12)	0.0457 (12)	0.0000 (9)	−0.0211 (9)	−0.0096 (10)
C27	0.0350 (10)	0.0329 (10)	0.0317 (10)	−0.0004 (8)	−0.0112 (8)	−0.0075 (8)
C28	0.0239 (9)	0.0294 (9)	0.0328 (10)	0.0044 (7)	−0.0077 (8)	−0.0086 (8)
C29	0.0339 (10)	0.0343 (10)	0.0336 (10)	0.0046 (8)	−0.0097 (8)	−0.0112 (8)
C30	0.0337 (10)	0.0417 (11)	0.0306 (10)	0.0069 (9)	−0.0050 (8)	−0.0069 (9)
C31	0.0373 (11)	0.0367 (11)	0.0401 (12)	−0.0019 (9)	−0.0065 (9)	−0.0007 (9)
C32	0.0477 (12)	0.0345 (11)	0.0498 (13)	−0.0036 (9)	−0.0161 (10)	−0.0090 (10)
C33	0.0354 (10)	0.0367 (11)	0.0337 (11)	0.0015 (8)	−0.0090 (8)	−0.0104 (9)

### 2-Methyl-1,1,2,3,3-pentaphenyl-2-silapropane (I) Geometric parameters (Å, º)

**Table:**

Si1—C1	1.8669 (18)	C15—H15	0.9500
Si1—C2	1.9099 (18)	C16—C17	1.388 (3)
Si1—C3	1.9138 (18)	C16—C21	1.387 (3)
Si1—C4	1.8744 (19)	C17—H17	0.9500
C1—H1A	0.9800	C17—C18	1.384 (3)
C1—H1B	0.9800	C18—H18	0.9500
C1—H1C	0.9800	C18—C19	1.380 (3)
C2—H2	1.0000	C19—H19	0.9500
C2—C10	1.526 (2)	C19—C20	1.376 (3)
C2—C16	1.524 (2)	C20—H20	0.9500
C3—H3	1.0000	C20—C21	1.388 (3)
C3—C22	1.527 (2)	C21—H21	0.9500
C3—C28	1.520 (2)	C22—C23	1.395 (2)
C4—C5	1.393 (3)	C22—C27	1.388 (3)
C4—C9	1.396 (3)	C23—H23	0.9500
C5—H5	0.9500	C23—C24	1.382 (3)
C5—C6	1.376 (3)	C24—H24	0.9500
C6—H6	0.9500	C24—C25	1.381 (3)
C6—C7	1.369 (3)	C25—H25	0.9500
C7—H7	0.9500	C25—C26	1.376 (3)
C7—C8	1.377 (3)	C26—H26	0.9500
C8—H8	0.9500	C26—C27	1.388 (3)
C8—C9	1.389 (3)	C27—H27	0.9500
C9—H9	0.9500	C28—C29	1.394 (3)
C10—C11	1.394 (2)	C28—C33	1.390 (3)
C10—C15	1.392 (3)	C29—H29	0.9500
C11—H11	0.9500	C29—C30	1.384 (3)
C11—C12	1.387 (3)	C30—H30	0.9500
C12—H12	0.9500	C30—C31	1.378 (3)
C12—C13	1.377 (3)	C31—H31	0.9500
C13—H13	0.9500	C31—C32	1.379 (3)
C13—C14	1.380 (3)	C32—H32	0.9500
C14—H14	0.9500	C32—C33	1.386 (3)
C14—C15	1.385 (3)	C33—H33	0.9500
			
C1—Si1—C2	111.34 (8)	C10—C15—H15	119.6
C1—Si1—C3	107.30 (9)	C14—C15—C10	120.79 (18)
C1—Si1—C4	109.22 (9)	C14—C15—H15	119.6
C2—Si1—C3	111.98 (8)	C17—C16—C2	118.87 (16)
C4—Si1—C2	106.03 (8)	C21—C16—C2	123.72 (16)
C4—Si1—C3	110.98 (8)	C21—C16—C17	117.42 (17)
Si1—C1—H1A	109.5	C16—C17—H17	119.4
Si1—C1—H1B	109.5	C18—C17—C16	121.26 (19)
Si1—C1—H1C	109.5	C18—C17—H17	119.4
H1A—C1—H1B	109.5	C17—C18—H18	119.7
H1A—C1—H1C	109.5	C19—C18—C17	120.5 (2)
H1B—C1—H1C	109.5	C19—C18—H18	119.7
Si1—C2—H2	105.2	C18—C19—H19	120.5
C10—C2—Si1	109.56 (11)	C20—C19—C18	119.09 (19)
C10—C2—H2	105.2	C20—C19—H19	120.5
C16—C2—Si1	117.65 (12)	C19—C20—H20	119.9
C16—C2—H2	105.2	C19—C20—C21	120.20 (19)
C16—C2—C10	112.90 (14)	C21—C20—H20	119.9
Si1—C3—H3	105.4	C16—C21—C20	121.47 (18)
C22—C3—Si1	116.07 (12)	C16—C21—H21	119.3
C22—C3—H3	105.4	C20—C21—H21	119.3
C28—C3—Si1	107.51 (11)	C23—C22—C3	119.13 (16)
C28—C3—H3	105.4	C27—C22—C3	123.19 (16)
C28—C3—C22	116.09 (14)	C27—C22—C23	117.65 (16)
C5—C4—Si1	120.97 (15)	C22—C23—H23	119.3
C5—C4—C9	116.66 (18)	C24—C23—C22	121.36 (18)
C9—C4—Si1	122.36 (15)	C24—C23—H23	119.3
C4—C5—H5	119.0	C23—C24—H24	119.9
C6—C5—C4	122.0 (2)	C25—C24—C23	120.21 (18)
C6—C5—H5	119.0	C25—C24—H24	119.9
C5—C6—H6	119.9	C24—C25—H25	120.4
C7—C6—C5	120.3 (2)	C26—C25—C24	119.18 (18)
C7—C6—H6	119.9	C26—C25—H25	120.4
C6—C7—H7	120.2	C25—C26—H26	119.6
C6—C7—C8	119.7 (2)	C25—C26—C27	120.72 (19)
C8—C7—H7	120.2	C27—C26—H26	119.6
C7—C8—H8	120.0	C22—C27—H27	119.6
C7—C8—C9	120.0 (2)	C26—C27—C22	120.87 (17)
C9—C8—H8	120.0	C26—C27—H27	119.6
C4—C9—H9	119.3	C29—C28—C3	122.69 (16)
C8—C9—C4	121.4 (2)	C33—C28—C3	119.78 (16)
C8—C9—H9	119.3	C33—C28—C29	117.43 (17)
C11—C10—C2	121.61 (16)	C28—C29—H29	119.4
C15—C10—C2	120.62 (16)	C30—C29—C28	121.16 (18)
C15—C10—C11	117.78 (17)	C30—C29—H29	119.4
C10—C11—H11	119.4	C29—C30—H30	119.9
C12—C11—C10	121.14 (18)	C31—C30—C29	120.25 (19)
C12—C11—H11	119.4	C31—C30—H30	119.9
C11—C12—H12	119.8	C30—C31—H31	120.1
C13—C12—C11	120.32 (18)	C30—C31—C32	119.74 (18)
C13—C12—H12	119.8	C32—C31—H31	120.1
C12—C13—H13	120.4	C31—C32—H32	120.1
C12—C13—C14	119.21 (18)	C31—C32—C33	119.76 (19)
C14—C13—H13	120.4	C33—C32—H32	120.1
C13—C14—H14	119.6	C28—C33—H33	119.2
C13—C14—C15	120.76 (19)	C32—C33—C28	121.64 (19)
C15—C14—H14	119.6	C32—C33—H33	119.2

### 2-Methyl-1,1,2,3,3-pentaphenyl-2-silapropane (I) Hydrogen-bond geometry (Å, º)

*Cg*2 and *Cg*4 are the centroids of the C10–C15 and C22–C27 rings, respectively.

**Table:**

*D*—H···*A*	*D*—H	H···*A*	*D*···*A*	*D*—H···*A*
C20—H20···*Cg*4^i^	0.95	2.94	3.716 (2)	140
C24—H24···*Cg*2^ii^	0.95	2.75	3.696 (2)	175

Symmetry codes: (i) −*x*+2, −*y*, −*z*; (ii) −*x*+2, −*y*+1, −*z*.

### 2-Methyl-1,1,3,3-tetraphenyl-2-silapropan-2-ol (II) Crystal data

**Table:**

C_27_H_26_OSi	*F*(000) = 840
*M**_r_* = 394.57	*D*_x_ = 1.231 Mg m^−^^3^
Monoclinic, *P*2_1_/*c*	Cu *K*α radiation, λ = 1.54178 Å
*a* = 11.8576 (5) Å	Cell parameters from 1512 reflections
*b* = 13.2995 (6) Å	θ = 4.0–71.6°
*c* = 14.3948 (6) Å	µ = 1.08 mm^−^^1^
β = 110.363 (3)°	*T* = 173 K
*V* = 2128.20 (16) Å^3^	Block, colourless
*Z* = 4	0.10 × 0.09 × 0.08 mm

### 2-Methyl-1,1,3,3-tetraphenyl-2-silapropan-2-ol (II) Data collection

**Table:**

Bruker APEXII CCD diffractometer	2414 reflections with *I* > 2σ(*I*)
φ and ω scans	*R*_int_ = 0.096
Absorption correction: multi-scan (SADABS; Bruker, 2013	θ_max_ = 72.1°, θ_min_ = 4.0°
*T*_min_ = 0.624, *T*_max_ = 0.754	*h* = −14→14
11901 measured reflections	*k* = −14→16
4113 independent reflections	*l* = −17→16

### 2-Methyl-1,1,3,3-tetraphenyl-2-silapropan-2-ol (II) Refinement

**Table:**

Refinement on *F*^2^	Primary atom site location: structure-invariant direct methods
Least-squares matrix: full	Secondary atom site location: difference Fourier map
*R*[*F*^2^ > 2σ(*F*^2^)] = 0.062	Hydrogen site location: mixed
*wR*(*F*^2^) = 0.171	H atoms treated by a mixture of independent and constrained refinement
*S* = 0.97	*w* = 1/[σ^2^(*F*_o_^2^) + (0.0793*P*)^2^] where *P* = (*F*_o_^2^ + 2*F*_c_^2^)/3
4113 reflections	(Δ/σ)_max_ = 0.001
267 parameters	Δρ_max_ = 0.29 e Å^−^^3^
0 restraints	Δρ_min_ = −0.35 e Å^−^^3^

### 2-Methyl-1,1,3,3-tetraphenyl-2-silapropan-2-ol (II) Special details

**Table:**

Geometry. All esds (except the esd in the dihedral angle between two l.s. planes) are estimated using the full covariance matrix. The cell esds are taken into account individually in the estimation of esds in distances, angles and torsion angles; correlations between esds in cell parameters are only used when they are defined by crystal symmetry. An approximate (isotropic) treatment of cell esds is used for estimating esds involving l.s. planes.

### 2-Methyl-1,1,3,3-tetraphenyl-2-silapropan-2-ol (II) Fractional atomic coordinates and isotropic or equivalent isotropic displacement parameters (Å^2^)

**Table:**

	*x*	*y*	*z*	*U*_iso_*/*U*_eq_	
Si1	0.37978 (7)	0.60730 (7)	0.11066 (6)	0.0322 (2)	
O1	0.4586 (2)	0.5078 (2)	0.0982 (2)	0.0478 (7)	
H1	0.525 (6)	0.511 (5)	0.125 (5)	0.14 (3)*	
C1	0.4582 (3)	0.7247 (3)	0.1046 (3)	0.0496 (10)	
H1A	0.538510	0.722512	0.152061	0.074*	
H1B	0.415753	0.780473	0.119230	0.074*	
H1C	0.461428	0.732630	0.039250	0.074*	
C2	0.2251 (3)	0.5965 (3)	0.0102 (2)	0.0311 (7)	
H2	0.186321	0.541154	0.032661	0.037*	
C3	0.3493 (3)	0.6018 (3)	0.2320 (2)	0.0305 (7)	
H3	0.304983	0.663543	0.233789	0.037*	
C4	0.2310 (3)	0.5599 (3)	−0.0879 (2)	0.0314 (7)	
C5	0.2221 (3)	0.4579 (3)	−0.1059 (3)	0.0465 (9)	
H5	0.212646	0.415105	−0.058001	0.056*	
C6	0.2269 (4)	0.4173 (3)	−0.1928 (3)	0.0597 (12)	
H6	0.220922	0.348044	−0.202343	0.072*	
C7	0.2403 (4)	0.4783 (4)	−0.2651 (3)	0.0594 (12)	
H7	0.242726	0.451111	−0.323899	0.071*	
C8	0.2500 (4)	0.5796 (4)	−0.2490 (3)	0.0558 (11)	
H8	0.259536	0.621704	−0.297355	0.067*	
C9	0.2457 (3)	0.6210 (3)	−0.1611 (3)	0.0446 (9)	
H9	0.252840	0.690203	−0.151519	0.054*	
C10	0.1461 (3)	0.6868 (3)	0.0079 (2)	0.0318 (7)	
C11	0.1675 (3)	0.7822 (3)	−0.0220 (3)	0.0402 (8)	
H11	0.233780	0.792719	−0.041198	0.048*	
C12	0.0917 (3)	0.8616 (3)	−0.0236 (3)	0.0478 (9)	
H12	0.106084	0.924134	−0.046117	0.057*	
C13	−0.0053 (3)	0.8494 (3)	0.0078 (3)	0.0495 (10)	
H13	−0.055857	0.903091	0.006978	0.059*	
C14	−0.0253 (3)	0.7557 (3)	0.0404 (3)	0.0463 (9)	
H14	−0.089227	0.746580	0.062864	0.056*	
C15	0.0485 (3)	0.6755 (3)	0.0402 (2)	0.0379 (8)	
H15	0.032870	0.612895	0.061775	0.046*	
C16	0.4654 (3)	0.6075 (3)	0.3226 (2)	0.0330 (7)	
C17	0.5172 (3)	0.7002 (3)	0.3555 (3)	0.0417 (9)	
H17	0.482228	0.758048	0.321262	0.050*	
C18	0.6206 (3)	0.7080 (3)	0.4391 (3)	0.0517 (10)	
H18	0.655291	0.770730	0.459002	0.062*	
C19	0.6718 (3)	0.6238 (3)	0.4923 (3)	0.0483 (10)	
H19	0.739662	0.629258	0.549267	0.058*	
C20	0.6213 (3)	0.5311 (3)	0.4603 (3)	0.0485 (10)	
H20	0.656040	0.473577	0.495283	0.058*	
C21	0.5185 (3)	0.5229 (3)	0.3757 (3)	0.0402 (8)	
H21	0.485335	0.459927	0.354838	0.048*	
C22	0.2661 (3)	0.5165 (3)	0.2345 (2)	0.0330 (7)	
C23	0.1654 (3)	0.5350 (3)	0.2610 (2)	0.0401 (8)	
H23	0.151976	0.599365	0.280198	0.048*	
C24	0.0848 (3)	0.4581 (3)	0.2589 (3)	0.0503 (10)	
H24	0.018734	0.471259	0.277708	0.060*	
C25	0.1022 (3)	0.3627 (3)	0.2294 (3)	0.0507 (11)	
H25	0.047234	0.311931	0.226694	0.061*	
C26	0.2021 (4)	0.3430 (3)	0.2038 (3)	0.0459 (9)	
H26	0.214929	0.278564	0.184382	0.055*	
C27	0.2826 (3)	0.4188 (3)	0.2069 (2)	0.0401 (8)	
H27	0.349762	0.404301	0.190084	0.048*	

### 2-Methyl-1,1,3,3-tetraphenyl-2-silapropan-2-ol (II) Atomic displacement parameters (Å^2^)

**Table:**

	*U*^11^	*U*^22^	*U*^33^	*U*^12^	*U*^13^	*U*^23^
Si1	0.0319 (4)	0.0348 (5)	0.0275 (4)	0.0005 (4)	0.0074 (3)	0.0024 (4)
O1	0.0406 (14)	0.0544 (18)	0.0440 (15)	0.0134 (13)	0.0092 (12)	−0.0039 (13)
C1	0.056 (2)	0.052 (3)	0.0361 (19)	−0.0172 (19)	0.0088 (17)	0.0081 (19)
C2	0.0329 (15)	0.0312 (18)	0.0273 (15)	−0.0035 (14)	0.0080 (12)	0.0031 (15)
C3	0.0369 (15)	0.0296 (17)	0.0250 (15)	0.0011 (14)	0.0109 (13)	−0.0004 (14)
C4	0.0295 (14)	0.0357 (19)	0.0259 (15)	0.0000 (14)	0.0057 (12)	−0.0012 (15)
C5	0.057 (2)	0.038 (2)	0.044 (2)	0.0052 (18)	0.0170 (18)	−0.0050 (18)
C6	0.064 (3)	0.048 (3)	0.065 (3)	0.000 (2)	0.020 (2)	−0.020 (2)
C7	0.051 (2)	0.075 (3)	0.052 (2)	−0.009 (2)	0.018 (2)	−0.030 (3)
C8	0.061 (2)	0.074 (3)	0.036 (2)	−0.016 (2)	0.0224 (18)	−0.008 (2)
C9	0.056 (2)	0.043 (2)	0.0346 (18)	−0.0080 (18)	0.0161 (16)	−0.0049 (18)
C10	0.0341 (15)	0.0340 (19)	0.0224 (15)	0.0024 (14)	0.0035 (12)	0.0004 (14)
C11	0.0462 (18)	0.034 (2)	0.043 (2)	0.0005 (16)	0.0191 (16)	0.0010 (17)
C12	0.057 (2)	0.033 (2)	0.053 (2)	0.0039 (17)	0.0201 (19)	0.0022 (19)
C13	0.053 (2)	0.040 (2)	0.052 (2)	0.0144 (18)	0.0137 (19)	−0.0072 (19)
C14	0.0429 (19)	0.051 (3)	0.047 (2)	0.0037 (17)	0.0176 (17)	−0.0020 (19)
C15	0.0401 (17)	0.036 (2)	0.0361 (18)	0.0022 (15)	0.0110 (14)	0.0023 (16)
C16	0.0344 (15)	0.042 (2)	0.0239 (14)	0.0003 (15)	0.0117 (12)	0.0022 (15)
C17	0.0471 (19)	0.039 (2)	0.0362 (18)	−0.0033 (16)	0.0111 (15)	−0.0015 (17)
C18	0.052 (2)	0.060 (3)	0.038 (2)	−0.018 (2)	0.0100 (17)	−0.009 (2)
C19	0.0381 (17)	0.073 (3)	0.0287 (17)	−0.0061 (19)	0.0054 (14)	0.001 (2)
C20	0.0407 (18)	0.063 (3)	0.0373 (19)	0.0030 (19)	0.0075 (16)	0.012 (2)
C21	0.0386 (17)	0.043 (2)	0.0353 (18)	−0.0004 (16)	0.0085 (15)	0.0046 (17)
C22	0.0366 (16)	0.0341 (19)	0.0239 (15)	−0.0030 (14)	0.0051 (13)	0.0058 (15)
C23	0.0424 (18)	0.045 (2)	0.0290 (17)	0.0017 (16)	0.0072 (14)	0.0034 (16)
C24	0.0377 (18)	0.070 (3)	0.039 (2)	−0.0073 (19)	0.0075 (16)	0.009 (2)
C25	0.052 (2)	0.052 (3)	0.0356 (19)	−0.0222 (19)	−0.0008 (17)	0.0107 (18)
C26	0.063 (2)	0.033 (2)	0.0337 (18)	−0.0076 (18)	0.0068 (17)	0.0041 (17)
C27	0.0480 (19)	0.037 (2)	0.0341 (18)	0.0006 (16)	0.0128 (15)	0.0073 (16)

### 2-Methyl-1,1,3,3-tetraphenyl-2-silapropan-2-ol (II) Geometric parameters (Å, º)

**Table:**

Si1—O1	1.666 (3)	C12—H12	0.9300
Si1—C1	1.835 (4)	C12—C13	1.384 (5)
Si1—C2	1.905 (3)	C13—H13	0.9300
Si1—C3	1.904 (3)	C13—C14	1.380 (5)
O1—H1	0.75 (6)	C14—H14	0.9300
C1—H1A	0.9600	C14—C15	1.380 (5)
C1—H1B	0.9600	C15—H15	0.9300
C1—H1C	0.9600	C16—C17	1.385 (5)
C2—H2	0.9800	C16—C21	1.383 (5)
C2—C4	1.518 (4)	C17—H17	0.9300
C2—C10	1.517 (4)	C17—C18	1.391 (5)
C3—H3	0.9800	C18—H18	0.9300
C3—C16	1.533 (4)	C18—C19	1.374 (5)
C3—C22	1.513 (4)	C19—H19	0.9300
C4—C5	1.378 (5)	C19—C20	1.378 (6)
C4—C9	1.389 (5)	C20—H20	0.9300
C5—H5	0.9300	C20—C21	1.396 (5)
C5—C6	1.382 (5)	C21—H21	0.9300
C6—H6	0.9300	C22—C23	1.396 (5)
C6—C7	1.372 (6)	C22—C27	1.392 (5)
C7—H7	0.9300	C23—H23	0.9300
C7—C8	1.366 (6)	C23—C24	1.394 (5)
C8—H8	0.9300	C24—H24	0.9300
C8—C9	1.397 (5)	C24—C25	1.376 (6)
C9—H9	0.9300	C25—H25	0.9300
C10—C11	1.391 (5)	C25—C26	1.383 (6)
C10—C15	1.397 (4)	C26—H26	0.9300
C11—H11	0.9300	C26—C27	1.378 (5)
C11—C12	1.381 (5)	C27—H27	0.9300
			
O1—Si1—C1	111.00 (17)	C11—C12—H12	119.5
O1—Si1—C2	106.65 (15)	C11—C12—C13	121.0 (4)
O1—Si1—C3	111.15 (15)	C13—C12—H12	119.5
C1—Si1—C2	113.46 (16)	C12—C13—H13	120.7
C1—Si1—C3	109.71 (16)	C14—C13—C12	118.5 (4)
C3—Si1—C2	104.69 (14)	C14—C13—H13	120.7
Si1—O1—H1	116 (5)	C13—C14—H14	119.6
Si1—C1—H1A	109.5	C15—C14—C13	120.8 (4)
Si1—C1—H1B	109.5	C15—C14—H14	119.6
Si1—C1—H1C	109.5	C10—C15—H15	119.4
H1A—C1—H1B	109.5	C14—C15—C10	121.1 (3)
H1A—C1—H1C	109.5	C14—C15—H15	119.4
H1B—C1—H1C	109.5	C17—C16—C3	119.7 (3)
Si1—C2—H2	104.2	C21—C16—C3	122.1 (3)
C4—C2—Si1	112.7 (2)	C21—C16—C17	118.2 (3)
C4—C2—H2	104.2	C16—C17—H17	119.5
C10—C2—Si1	112.4 (2)	C16—C17—C18	121.0 (4)
C10—C2—H2	104.2	C18—C17—H17	119.5
C10—C2—C4	117.5 (3)	C17—C18—H18	119.8
Si1—C3—H3	105.5	C19—C18—C17	120.5 (4)
C16—C3—Si1	112.2 (2)	C19—C18—H18	119.8
C16—C3—H3	105.5	C18—C19—H19	120.5
C22—C3—Si1	112.6 (2)	C18—C19—C20	119.1 (3)
C22—C3—H3	105.5	C20—C19—H19	120.5
C22—C3—C16	114.6 (3)	C19—C20—H20	119.7
C5—C4—C2	117.7 (3)	C19—C20—C21	120.5 (4)
C5—C4—C9	117.1 (3)	C21—C20—H20	119.7
C9—C4—C2	125.2 (3)	C16—C21—C20	120.7 (4)
C4—C5—H5	119.0	C16—C21—H21	119.7
C4—C5—C6	122.0 (4)	C20—C21—H21	119.7
C6—C5—H5	119.0	C23—C22—C3	120.1 (3)
C5—C6—H6	119.7	C27—C22—C3	122.4 (3)
C7—C6—C5	120.5 (4)	C27—C22—C23	117.5 (3)
C7—C6—H6	119.7	C22—C23—H23	119.7
C6—C7—H7	120.6	C24—C23—C22	120.7 (4)
C8—C7—C6	118.7 (4)	C24—C23—H23	119.7
C8—C7—H7	120.6	C23—C24—H24	119.7
C7—C8—H8	119.5	C25—C24—C23	120.5 (4)
C7—C8—C9	121.0 (4)	C25—C24—H24	119.7
C9—C8—H8	119.5	C24—C25—H25	120.3
C4—C9—C8	120.7 (4)	C24—C25—C26	119.5 (4)
C4—C9—H9	119.7	C26—C25—H25	120.3
C8—C9—H9	119.7	C25—C26—H26	120.0
C11—C10—C2	123.5 (3)	C27—C26—C25	120.0 (4)
C11—C10—C15	117.5 (3)	C27—C26—H26	120.0
C15—C10—C2	119.0 (3)	C22—C27—H27	119.1
C10—C11—H11	119.5	C26—C27—C22	121.9 (4)
C12—C11—C10	121.0 (3)	C26—C27—H27	119.1
C12—C11—H11	119.5		

### 2-Methyl-1,1,3,3-tetraphenyl-2-silapropan-2-ol (II) Hydrogen-bond geometry (Å, º)

*Cg*1 is the centroid of the C4–C9 ring.

**Table:**

*D*—H···*A*	*D*—H	H···*A*	*D*···*A*	*D*—H···*A*
C27—H27···O1	0.93	2.55	3.237 (4)	131
O1—H1···*Cg*1^i^	0.75 (8)	2.70 (7)	3.416 (3)	162 (7)
C24—H24···*Cg*1^ii^	0.93	2.86	3.582 (4)	135

Symmetry codes: (i) −*x*+1, −*y*+1, −*z*; (ii) −*x*, −*y*+1, −*z*.
